# Supplementary material for: The interactome of intact mitochondria by cross-linking mass spectrometry provides evidence for coexisting respiratory supercomplexes
Source: Mol Cell Proteomics. 2017 Dec 8;17(2):216–32. doi: 10.1074/mcp.RA117.000470 (PMC5795388; doi:10.1074/mcp.RA117.000470)
Supplement: Supplemental Data [file supp_RA117.000470_133922_0_supp_23978_fzffwf.zip › spectra_annotation/mito_spectra_annotation/226-1-9-1-11-1.pdf]

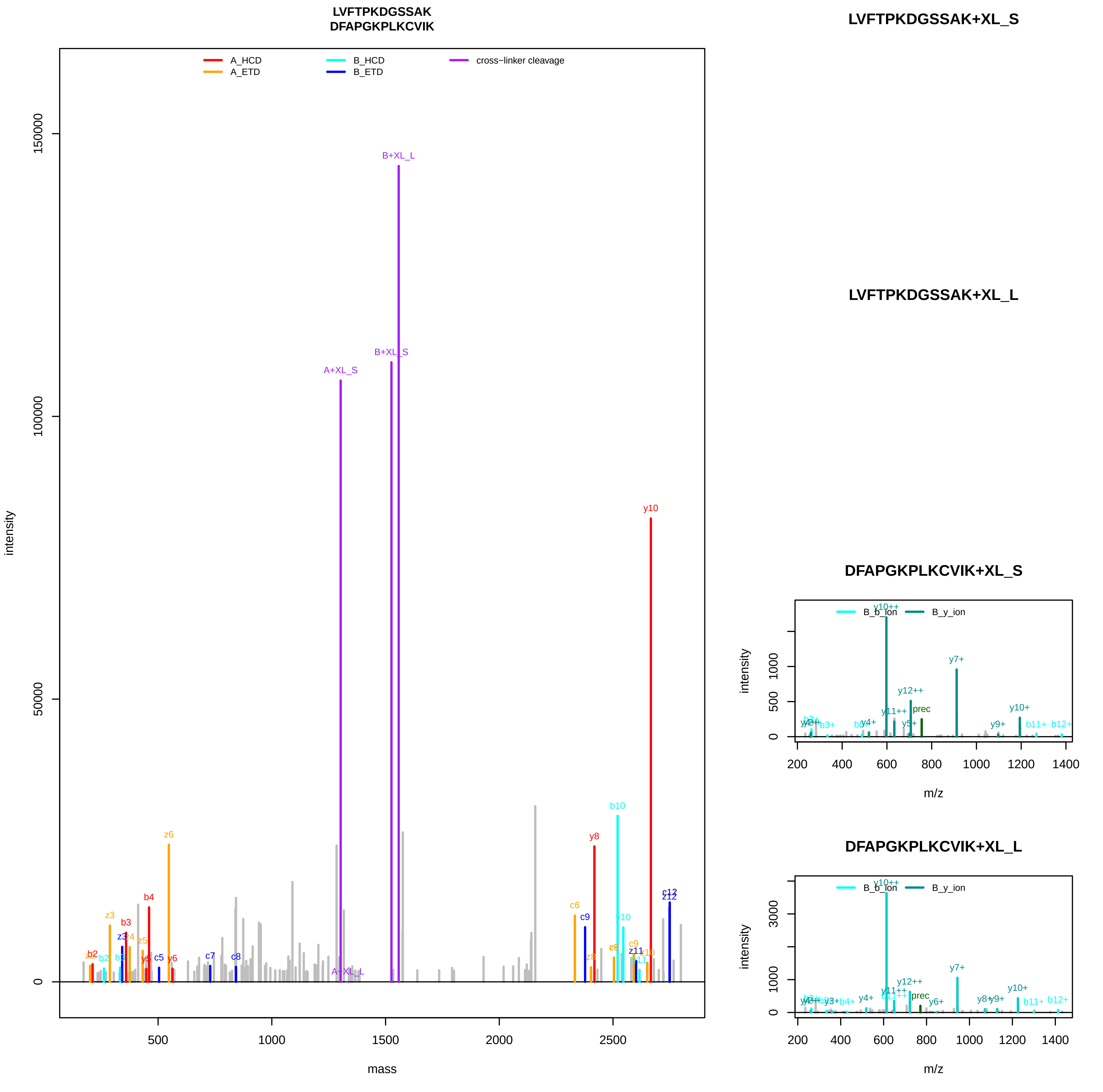

LVFTPKDGSSAK
DFAPGKPLKCVIK
A_HCD
A_ETD
B_HCD
B_ETD
cross−linker cleavage
intensity
mass
0
50000
100000
150000
500
1000
1500
2000
2500
B+XL_L
B+XL_S
A+XL_S
A+XL_L
y10
b10
y8
z6
c6
c9
c12
z12
y10
b4
z3
b3
z3
z4
z5
b2
b2
c5
y6
c7
c8
z8
z9
c9
z10
z11
y11
LVFTPKDGSSAK+XL_S
LVFTPKDGSSAK+XL_L
DFAPGKPLKCVIK+XL_S
B_b_ion
B_y_ion
y10++
y7+
y12++
prec
y11++
y5+
y4+
b3+
y9+
y10+
b11+
b12+
m/z
200
400
600
800
1000
1200
1400
0
500
1000
DFAPGKPLKCVIK+XL_L
B_b_ion
B_y_ion
y10++
y7+
y12++
y11++
b11++
prec
y6+
y4+
b4+
y3+
y8+
y9+
y10+
b11+
b12+
m/z
1000
3000
